# Hypothyroidism among military infants born in countries of varied iodine nutrition status

**DOI:** 10.1186/1472-6823-10-2

**Published:** 2010-02-01

**Authors:** Marcus M Cranston, Margaret AK Ryan, Tyler C Smith, Carter J Sevick, Stephanie K Brodine

**Affiliations:** 1 Keesler Medical Center, Keesler AFB, Mississippi, USA; 2 Naval Hospital, Camp Pendleton, California, USA; 3 Naval Health Research Center, San Diego, California, USA; 4 San Diego State University, San Diego, California, USA

## Abstract

**Background:**

Iodine deficiency is a global problem representing the most common preventable cause of mental retardation. Recently, the impact of subtle deficiencies in iodine intake on children and pregnant women has been questioned. This study was designed to compare hypothyroidism among infants born to US military families in countries of varied iodine nutrition status.

**Methods:**

A cohort design was used to analyze data from the Department of Defense Birth and Infant Health Registry for infants born in 2000-04 (*n *= 447,691). Hypothyroidism was defined using ICD-9-CM codes from the first year of life (*n *= 698). The impact of birth location on hypothyroidism was assessed by comparing rates in Germany, Japan, and US territories with the United States, while controlling for infant gender, plurality, gestational age, maternal age, maternal military status, and military parent's race/ethnicity.

**Results:**

Hypothyroidism did not vary by birth location with adjusted odds ratios (OR) as follows: Germany (OR 0.82, [95% CI 0.50, 1.35]), Japan (OR 0.67, [95% CI 0.37, 1.22]), and US territories (OR 1.29, [95% CI 0.57, 2.89]). Hypothyroidism was strongly associated with preterm birth (OR 5.44, [95% CI 4.60, 6.42]). Hypothyroidism was also increased among infants with civilian mothers (OR 1.24, [95% CI 1.00, 1.54]), and older mothers, especially ages 40 years and older (OR 2.09, [95% CI 1.33, 3.30]).

**Conclusions:**

In this study, hypothyroidism in military-dependent infants did not vary by birth location, but was associated with other risk factors, including preterm birth, civilian maternal status, and advanced maternal age.

## Background

Over 1.9 billion people, 31% of the world's population, live in areas of iodine deficiency [1]. Iodine deficiency disorders affect over 200 million people and are the most common preventable cause of brain damage and mental retardation worldwide [2]. Iodine deficiency exists primarily in areas of low soil iodine content, and although strategies exist to combat iodine deficiency, they are not employed uniformly throughout the world [3]. As a result, iodine nutrition varies between countries.

Early public health efforts focused on severe iodine deficiency with the goal of preventing goiter and cretinism. More recently, concerns have been raised regarding the effects of lesser degrees of iodine deficiency in vulnerable populations, such as pregnant women and children. When iodine deficiency is present during the fetal stage to the third month after birth, even mild to moderate deficiency may lead to abnormalities of psychoneuromotor and intellectual development [4,5].

Iodine deficiency disorders were a significant problem in the United States until the 1920s, when the general use of iodized salt was initiated [6]. Based on the National Health and Nutrition Examination Survey (NHANES) III, performed from 1988 to 1994, the World Health Organization (WHO) has classified US iodine intake as "more than adequate" [3]. However, the adequacy of iodine intake during pregnancy has been questioned, as changes in thyroid hormone metabolism and renal iodine handling increase maternal iodine needs [4,7]. It has been proposed that a median urinary iodine (UI) concentration of 150-245 ug/l during pregnancy be used to define a population with sufficient iodine intake [8]. NHANES III and the 2001-2002 NHANES reported median UI concentrations at the lower limit of these recommendations for pregnancy, 141 and 173 ug/l, respectively [7,9].

The overwhelming majority of hypothyroidism during infancy is due to congenital hypothyroidism with etiologies including a variety of environmental and genetic factors, but the major determinant of prevalence worldwide is iodine intake. In areas of iodine sufficiency, congenital hypothyroidism may be as rare as 1:4000, while transient thyrotropin elevations may be seen in up to 40% of neonates in areas of severe iodine deficiency [1,10]. The impact of more subtle differences in iodine intake on hypothyroidism is unclear.

Families of US military members often reside in countries with varied iodine nutrition status. The majority of military-dependent births outside of the United States occur in Germany where iodine intake in the host-nation population is lower than the United States, Japan where iodine intake is higher than the United States and US territories in which little data regarding iodine intake is available [3,11]. No prior studies have examined iodine intake, iodine nutrition status or iodine-dependent outcomes in US military families residing in these different geographic locations. However, all infants sponsored by the Department of Defense (DoD) undergo hypothyroidism screening at birth, and diagnostic data for these children are captured in the DoD Birth and Infant Health Registry, previously titled the DoD Birth Defects Registry. As a result, this registry provides a unique opportunity to compare rates of hypothyroidism during infancy and gain insight into the relative iodine nutrition of military families living in these areas. In addition, the DoD Birth and Infant Health Registry is positioned to examine other potential risk factors for hypothyroidism during infancy, an area in which prior studies have focused on congenital hypothyroidism and are somewhat limited. Therefore, this study was designed to compare hypothyroidism among infants born to US military families in countries where the host populations have varied iodine nutrition status.

## Methods

### Study population

This cohort study used the DoD Birth and Infant Health Registry to identify all infants born to US military members during 2000-2004. Potential subjects were excluded for incomplete gender data (*n *= 26) and maternal age (*n *= 8), as well as the inability to reliably link diagnosis data (*n *= 22). In addition, only infants born in the United States, Germany, Japan, or US territories, the locations with numbers of births sufficient for comparison, were included in this study (*n *= 447,691). This research has been conducted in compliance with all applicable federal regulations governing the protection of human subjects in research (Protocol NHRC.2007.0026).

### Identification of hypothyroidism

All military-sponsored infants undergo hypothyroidism screening, regardless of birth location, and the DoD Birth and Infant Health Registry captures all hospitalization and outpatient encounter data for military-dependent children during the first year of life using International Classification of Diseases, Clinical Modification (ICD-9-CM)-coded diagnoses. Further details regarding the registry have been previously published [12]. For this study, infant records were searched for ICD-9-CM codes 243, congenital hypothyroidism, and 244, acquired hypothyroidism [13]. To avoid inclusion of screening encounters in the outcome, cases of hypothyroidism were defined as those infants with the ICD-9-CM code 243 or 244 on at least two separate health care encounter dates, or at least one inpatient encounter.

### Hypothyroidism risk factors

The independent variable of interest was birth location, and the United States, Germany, Japan, and US territories represented the location of 96.8% of all military-dependent births. Births in the current and former US territories of American Samoa, Federated States of Micronesia, Guam, Marshall Islands, Northern Mariana Islands, Palau, Puerto Rico, US Virgin Islands, and other outlying islands were categorized as US territory. Variables previously shown to be associated with congenital hypothyroidism were also assessed, including maternal age, infant gender, infant plurality, and estimated gestational age [14-16]. The estimated gestational age in this study was classified as full-term (≥37 weeks) or preterm (<37 weeks), based on ICD-9-CM codes in 765 series. Infant race/ethnicity, which has been reported to be associated with congenital hypothyroidism, was not available [16]. However, the race/ethnicity of the military sponsor, the parent to whom health care eligibility is linked, was analyzed. This was the only independent variable with missing data and an unknown category was used where the race/ethnicity data for the military sponsor were not available (*n *= 14,786). White, non-Hispanic race/ethnicity was designated as the reference due to the previously reported lower rates of congenital hypothyroidism for infants in this category [16]. Maternal military status was categorized as active-duty military or civilian.

### Statistical methods

Univariate analyses were performed to explore the unadjusted associations of hypothyroidism, location of birth, and demographic characteristics. The impact of independent variables on hypothyroidism was estimated as crude and adjusted odds ratios with 95% confidence intervals by applying a multivariable logistic regression model. Regression diagnostics and multicollinearity investigations were completed. All independent variables were included in the final logistic regression model due to either significant unadjusted odds ratios or previously reported associations with congenital hypothyroidism. Computer analyses were performed using SAS version 9.1 (SAS Institute, Inc., Cary, North Carolina).

## Results

The characteristics of the study population of infants from the DoD Birth and Infant Health Registry born in 2000-2004 (*n *= 447,691) are illustrated in Table 1. These infants were predominately born in the United States to civilian mothers, who were married to active-duty military members. The majority of military sponsors self-reported their race/ethnicity to be white, non-Hispanic. The rate of preterm births was 8.8%, while 2.8% were multiple births.

**Table 1:** Characteristics of infants in the DoD Birth and Infant Health Registry, born 2000-2004 (*N *= 447 691)

Characteristic	*n *(%)	
Birth location		

United States	419 756	(93.8)

Germany	13 685	(3.1)

Japan	10 994	(2.5)

US territory	3256	(0.7)

Sex of infant		

Male	229 530	(51.3)

Female	218 161	(48.7)

Infant plurality		

Singleton	435 021	(97.2)

Multiple birth	12 670	(2.8)

Estimated gestational age (weeks)		

Full-term (≥37)	408 224	(91.2)

Preterm (<37)	39 467	(8.8)

Maternal age (years)		

≤19	27 951	(6.2)

20-24	164 066	(36.6)

25-29	129 000	(28.8)

30-34	85 433	(19.1)

35-39	34 742	(7.8)

≥40	6499	(1.5)

Maternal military status		

Civilian	365 953	(81.7)

Military	81 738	(18.3)

Military sponsor race/ethnicity		

White, non-Hispanic	287 834	(64.3)

Black, non-Hispanic	76 529	(17.1)

Hispanic	42 382	(9.5)

Asian/Pacific Islander	16 044	(3.6)

American Indian/Alaska Native	5510	(1.2)

Other	4606	(1.0)

Unknown	14 786	(3.3)

DoD, US Department of Defense

Odds ratios for the associations of hypothyroidism with birth location and other independent characteristics are shown in Table 2. Hypothyroidism did not differ for infants born in Germany (OR 0.82, [95% CI 0.50, 1.35]), Japan (OR 0.67, [95% CI 0.37, 1.22]) or US territories (OR 1.29, [95% CI 0.57, 2.89]) when compared with infants born in the United States. Hypothyroidism was associated with preterm birth (OR 5.44, [95% CI 4.60, 6.42]). In addition, hypothyroidism was more frequent among infants born to older mothers, women aged 25-29 years (OR 1.32, [95% CI 1.09, 1.59]) and women aged 40 years and older OR 2.09, [95% CI 1.33, 3.30]) when compared with infants with younger mothers, aged 20-24 years. Hypothyroidism was also slightly more common in infants born to civilian mothers (OR 1.24, [95% CI 1.00, 1.54]), than for those born to active-duty military mothers. Infant gender, plurality, and military sponsor race/ethnicity were not found to be associated with hypothyroidism. Of note, there was no significant change in odds ratio measures for birth locations when infants with missing data for military sponsor race/ethnicity were excluded from the analysis.

**Table 2:** Adjusted odds of congenital hypothyroidism for infants in DoD Birth and Infant Health Registry, born 2000-2004 (*N *= 447 691)

Variable	No Hypothyroidism (*N *= 446 993)	Hypothyroidism (*N *= 698)	**OR**^**a**^ [95% CI]
Birth location			

United States	419 091	665	1.00 Reference

Germany	13 669	16	0.82 [0.50, 1.35]

Japan	10 983	11	0.67 [0.37, 1.22]

US territory	3250	6	1.29 [0.57, 2.89]

Sex of infant			

Male	229 163	367	1.00 Reference

Female	217 830	331	0.97 [0.84, 1.13]

Infant plurality			

Singleton	434 375	646	1.00 Reference

Multiple Birth	12 618	52	0.91 [0.67, 1.23]

Estimated gestational age (weeks)			

Full-term (≥37)	407 765	459	1.00 Reference

Preterm (<37)	39 228	239	**5.44 [4.60, 6.42]**

Maternal age (years)			

≤19	27 911	40	1.04 [0.74, 1.45]

20-24	163 852	214	1.00 Reference

25-29	128 777	223	**1.32 [1.09, 1.59]**

30-34	85 298	135	1.16 [0.93, 1.44]

35-39	34 678	64	1.27 [0.95, 1.69]

≥40	6477	22	**2.09 [1.33, 3.30]**

Maternal military status			

Military	81 633	105	1.00 Reference

Civilian	365 360	593	**1.24 [1.00, 1.54]**

Military sponsor race/ethnicity			

White, non-Hispanic	287 401	433	1.00 Reference

Black, non-Hispanic	76 402	127	1.09 [0.89, 1.33]

Hispanic	42 321	61	1.01 [0.77, 1.33]

Asian/Pacific Islander	16 018	26	1.12 [0.75, 1.66]

American Indian/Alaska Native	5498	12	1.55 [0.87, 2.76]

Other	4600	6	0.88 [0.39, 1.98]

Unknown	14 753	33	1.26 [0.87, 1.81]

DoD, US Department of Defense; OR, adjusted odds ratio; CI, confidence interval.

^a^Model adjusted for birth location, sex of infant, infant plurality, estimated gestational age, maternal age, maternal military status, and military sponsor race/ethnicity.

## Discussion

In this study of infants born to US military families, our data did not demonstrate differences in hypothyroidism for the birth locations examined. Iodine intake has never been studied in these military family populations and the application of practices that may increase iodine intake, such as consuming US-based food products on military bases or iodine-containing prenatal vitamins, is unknown. For example, the Department of Defense uses multiple US-based sources for prenatal vitamins and during the period of this study, some of the sources contained iodine and others did not. With the lack of information regarding iodine intake among US military families, the findings of this study are reassuring given the historical differences in iodine intake and iodine deficiency disorders in the host nation populations in these regions of the world. In the 1980s, estimates of transient neonatal hypothyroidism in Belgium were as high as 1:100 compared with 1:50 000 in the United States [17]. Over the past two decades iodine intake has decreased in the United States and increased in Europe, but differences in iodine nutrition still exist and the WHO classification of iodine nutrition is lower for Germany than the United States [3,7]. In Japan there is an absence of nationally representative data, but available data suggest iodine intake is much higher than in the United States [11]. Specific iodine nutrition data are also lacking for the current and former US territories examined in this study, but WHO regional data would suggest that intake in these areas is significantly less than that of the United States [3]. The influence of host nation practices on the nutritional status of military families is not known and iodine-dependent outcomes have not previously been assessed in these populations, highlighting the importance of the findings of this study.

Given universal screening for congenital hypothyroidism at birth, along with the rare clinical recognition of acquired hypothyroidism due to indolent onset and nonspecific symptoms during infancy, the majority of cases of hypothyroidism in our study likely represent congenital hypothyroidism, despite provider use of both ICD-9-CM codes 243, congenital hypothyroidism, and 244, acquired hypothyroidism. This is supported by the fact that 76.1% of those with ICD-9-CM code 243 and 58.8% of those with ICD-9 code 244 had diagnostic codes entered within the first 10 weeks of life. Iodine deficiency is responsible for the majority of transient congenital hypothyroidism, while permanent congenital hypothyroidism causes include thyroid dysgenesis, dyshormonogenesis, and central hypothyroidism and is relatively rare, with incidence estimates for each etiology ranging from 1:4500 to 1:100 000 [18]. With the exception of inborn errors of thyroid hormone synthesis, the etiologic mechanism in most cases is unknown, thus contributing to the interest in identifying risk factors for congenital hypothyroidism [19].

In our study, the factor most strongly association with hypothyroidism was preterm birth. This is consistent with previous descriptive studies of congenital hypothyroidism and is thought to represent transient hypothyroxinemia, due to incomplete thyroid gland development [20,21]. We also found hypothyroidism during infancy to be associated with increased maternal age, with the greatest risk for those aged 40 years and older. Previously, a registry-based study of congenital hypothyroidism in California found no linear trend across maternal age categories, while an Italian case-control study found higher rates of permanent congenital hypothyroidism for maternal age ≥40 years [15,16]. Our study also found hypothyroidism to be slightly more common in infants born to civilian mothers. These associations between maternal factors and hypothyroidism may represent differences in the health of the maternal populations. It is possible that younger age and the military entrance screening process reduce the prevalence of maternal disorders, such as autoimmune diseases, and decrease the risk for genetic disorders, leading to lower rates of hypothyroidism among infants born to these maternal groups.

Interestingly, we did not find hypothyroidism to be associated with infant gender, while most prior studies of congenital hypothyroidism have shown a female predominance [15,16,20,22-24]. However, studies isolating permanent and transient causes have found a gender influence only among permanent cases of congenital hypothyroidism [15,20]. Given our use of ICD-9-CM codes to define cases, it is likely that many transient cases were captured. This is suggested by our relatively high overall prevalence and may explain the lack of a gender relationship in our study. Congenital hypothyroidism rates have been reported to be higher for Hispanic, Native American, and Asian infants and lower for black infants when compared to white infants [16,23,24]. However, we did not see an association with the ethnicity/race of the military sponsor, which may be due to an absence of data regarding the spouse.

While the DoD Birth and Infant Health Registry provides many advantages, certain limitations must be considered. Defining cases by ICD-9-CM code alone raises concern regarding the accuracy of the outcome measure. However, given that screening is universal, health care encounter data are captured electronically, the number of hypothyroidism codes is limited, and hypothyroidism care requires multiple clinic visits for the infant, unidentified cases of hypothyroidism should be rare in this study. In addition, we required diagnostic codes from two separate health care encounter dates to improve specificity, primarily by excluding visits to perform screening tests. The use of birth location as a measure of the geographically-defined iodine nutrition exposure also has potential limitations. However, given that thyroid function adapts to changes in iodine intake within weeks and travel during the final weeks of pregnancy is uncommon, birth location likely corresponds well with the location of iodine exposure that would impact thyroid function at the time of birth. Finally, the birth locations examined in this study were limited to those with the largest sample sizes and the possibility of the presence of locations of greater risk must be considered.

## Conclusions

Military families and other mobile populations assume health risks associated with their varied environments. Therefore, ongoing surveillance and studies are needed to detect adverse outcomes and determine the need for intervention. In this study, the DoD Birth and Infant Health Registry allowed for the first-ever investigation of an iodine-dependent outcome in a mobile population across different locations. While the assessment of other potential risk factors contributes to the overall understanding of hypothyroidism in infancy, the lack of differences in hypothyroidism rates for the geographic areas studied provides much-needed reassurance for military families and medical providers.

## Competing interests

The authors declare that they have no competing interests.

## Authors' contributions

MC, MR, TS, CS, and SB all helped conceive the study, participated in its design and coordination, and helped to draft the manuscript. All authors read and approved the final manuscript.

## Pre-publication history

The pre-publication history for this paper can be accessed here:

http://www.biomedcentral.com/1472-6823/10/2/prepub
